# The association between alteration of maternal lipid levels and birthweight at term: A within-family comparison

**DOI:** 10.3389/fendo.2022.989663

**Published:** 2022-09-30

**Authors:** Qinqing Chen, Huiqi Chen, Minmin Wang, Liping Qiu, Fangfang Xi, Ying Jiang, Min Lv, He-Feng Huang, Qiong Luo

**Affiliations:** ^1^ Department of Obstetrics, Women’s Hospital, School of Medicine, Zhejiang University, Hangzhou, China; ^2^ School of Public Health, Sun Yat-sen University, Guangzhou, China

**Keywords:** maternal lipid, body mass index, total cholesterol, birthweight, within-family comparison

## Abstract

**Context:**

Maternal lipid levels affect birthweight and the long-term health of the offsprings. However, this association could be influenced by genetic and other common factors.

**Objective:**

This work aimed to explore the relationship between maternal lipid levels and birthweight of two pregnancies in the same mother.

**Methods:**

In this population-based cohort study, 705 women and their 1 410 offsprings were included. From an initial sample of women with more than one singleton birth in the database, we made the following exclusions: missing data for pre-pregnancy BMI, pregnancy weight gain, birthweight and lipid values; maternal age less than 19 or older than 44 years old; gestational age < 37 weeks or > 41weeks, gestational diabetes mellitus/diabetic. In the second and third trimesters, serum samples were collected for the determination of fasting total cholesterol (TC), triglycerides (TG), high-density lipoprotein cholesterol (HDL-C), and low-density lipoprotein cholesterol (LDL-C) levels. Then we assessed the association between maternal lipids and birthweight.

**Results:**

Infants of women whose 2^nd^-trimester TC increased by 10th-20th percentile (-0.92~-0.56 mmol/L) from 1st to 2nd pregnancy were 239.69 (62.32~417.06) g lighter at birth than were infants of women those of 40th-50th percentile (-0.20~-0.03 mmol/L). Parity, gestational age, neonatal gender, maternal pre-pregnancy body mass index, maternal weight gain, and 3^rd^-trimester TC and HDL-C were all associated with higher birth weight. Every unit increase in TC in the third trimester increases birthweight by 53.13 (14.32 ~91.94) g.

**Conclusion:**

Maternal TC level is associated with birthweight independent of shared genes. TC may be used to guide diet and predict birthweight combined with ultrasound and other indicators.

## Introduction

Association between birthweight and fetal health in the short- and long-term has been confirmed. This fetal origin of adult disease was first proposed by Baker and colleagues (1) and has been confirmed by a large number of human studies. For instance, too low or too high infant birthweight both increase the risk for metabolic diseases in adulthood including obesity, hypertension, cardiovascular diseases, type 2 diabetes mellitus, insulin resistance and hyperlipidemia (2, 3). With the onset of the epidemic of metabolic diseases in offsprings, many experiments on animals have confirmed that maternal lipid metabolism disorders led to metabolic related diseases in offsprings (4, 5). Research also demonstrated that the effects of perinatal dyslipidemia would persist after birth (6).

During the normal gestational period, the level of blood lipid profiles including TG, TC, LDL-C and HDL-C gradually increase from the 12^th^ week of gestation and this increasing trend continues to the third trimester (7–9). These changes in lipids are needed to maintain the growth and development of the fetus. The two main changes in fat metabolism during pregnancy are fat accumulation and hyperlipidemia (4). Maternal TG levels in late pregnancy of women diagnosed with gestational diabetes mellitus (GDM) are found to have a significant positive correlation with neonatal birthweight, fat mass and body mass index (BMI) (10–12). One previous study in China showed that high TG and low HDL-C values in late pregnancy could be considered as predictors of a high risk of large for gestational age (LGA) and macrosomia, regardless of infant gender, which was coincident with our results (13). A study including women with normal glucose tolerance discovered that pre-pregnancy BMI and maternal TG values in the last trimester were associated with newborn birthweight independently (14). In a prospective population-based cohort study, higher maternal TG levels were positively correlated with higher birthweight and that was found in normal weight women only (15). It remains controversial whether the association between maternal lipid levels and newborn birthweight appears only in pregnant women with normal glucose or also in GDM/diabetic pregnancies Our previous study excluded pregnancies with relevant complications like GDM, diabetes, pre-eclampsia(PE) and concluded that high HDL-C levels during the third trimester were significantly associated with small for gestational age (SGA), independent of gestational weight gain (16).

Direct association between maternal lipid profiles during pregnancy and birthweight have generally been shown in previous studies. However, interindividual comparisons conducted in these observational studies have a major characteristic limitation from genetic and environmental confoundings. In a large population-based cohort study published in Lancet (17) examined how differences in weight gain during two or more pregnancies for the same mother predicted the birthweight of her offspring. Therefore in our study, we aimed to explore the association between maternal lipids and birthweight by comparing outcomes from two pregnancies of each woman. This within-family research was designed to reduce genetic confounding and other potential individual factors.

## Materials and methods

### Study design and population

Data for this retrospective study were from natality records covering all births in Women’s Hospital, Zhejiang University School of Medicine from 1 Jan 2014 and 31 Dec 2020. Records of birth outcomes, maternal characteristics and prenatal healthcare are available from the hospital information system. Children born to the same mother were recorded under the unique patient’s identification number and we have checked the fathers’ identification number to ensure children born to the same father.

We made the following exclusions from an initial sample of singleton births: missing data for pre-pregnancy BMI, pregnancy weight gain, birthweight and lipid values; maternal age less than 19 or older than 44 years old; gestational age < 37 weeks or > 41weeks; maternal diabetes; inherited metabolic diseases before pregnancy; malignant tumor and chromosomal abnormalities; consumed alcohol or drugs, used tobacco and systemic infection during pregnancy that may influence lipid metabolism; birthweight > 7000g or < 500g (extreme values that could come from data export error). The study was conducted under an approval from the hospital’s Clinical Research Ethics Committee.

### Data collection

All the data of birth outcomes, maternal characteristics and lipid values were obtained from the hospital information system of Women’s Hospital, Zhejiang University School of Medicine. The variables included were: gravidity, parity, maternal age, husband age, gestational age, education background, pre-pregnancy weight, height, pregnancy weight gain, child gender, and lipid values in the second and third trimester. Data for birth outcomes and maternal characteristics were recorded by midwives and checked with obstetricians, which made it highly reliable. Maternal pre-pregnancy BMI was calculated from pre-pregnancy height and weight, and categorized into overweight (≥25.0 kg/m^2^), normal weight (18.5-24.9 kg/m^2^), and underweight (<18.5 kg/m^2^) groups on the basis of the World Health Organization BMI classification (18).

Mothers retained in the sample were all maintained regular prenatal healthcare in our hospital and venous blood samples were taken from all the mothers after eight hours fasting at the second (24-26 gestational weeks) and third (30–32 gestational weeks) trimester of pregnancy. All the blood samples were assayed for LDL-C, HDL-C, TG and TC concentrations. An automatic biochemical analyzer was used to perform the measurements and the specific methods had been described in our previous study (16).

### Statistical analysis

We expected that the maternal TC level is positively associated with birthweight and HDL-C level has a negatively relationship with birthweight after adjustment for potential confounders as reported previously. Our inclusion and exclusion criteria were set up to maximum eliminate some sources of potential confounding, like gestational age, maternal diabetes, and extremes in birth weight. For residual confounding by measured (smoking) and unmeasured (shared genetic and environmental) covariates, we adopted the strategy of comparing two pregnancies in the same mother and including measured covariates in the statistical models. Thus, we kept the effects of differences between individuals to a minimum.

In the mixed-effects model, we take ID number of pregnant women as random effects terms, and maternal age, pre-pregnancy BMI, gestational weight gain, gestational age at birth, parity, infant gender, the interval of two pregnancies were regarded as fixed effects terms.

Linear regression analysis was applied to examine the associations between changes in maternal lipid values and changes in birthweight between two pregnancies for each mother. First, we classified the changes of maternal lipid values (TC, TG, HDL-C, LDL-C) by percentiles, from the minimum value (min) to maximum value (max). We then regressed changes in birthweight (continuous variable) on categories of changes in maternal serum levels (min-P10, P10-20, and so on, until P80-90, P90-max). In the multivariable adjusted model, maternal age, parity, gestational weight gain, pre-pregnancy BMI, gestational age at birth, infant gender, the interval of two pregnancies and the difference in gestational age were regarded as confounding variables. The variable values refer to the values of the second observed pregnancy minus the value of the first observed pregnancy for each mother.

All statistical analyses were conducted using the R software, version 4.0.1 for Windows (The R Project; https://www.r-project.org). P values < 0.05 were considered statistically significant. All data were presented as means and SDs for maternal cohort characteristics or 95% CIs for outcome data.

## Results

From an initial sample size of 2144 singleton births from 1072 mothers who had two records of birth from 1 Jan 2014 and 31 Dec 2020, exclusions were as follows: gestational diabetes mellitus (GDM)/diabetic (440births), gestational age < 37 weeks or >41 weeks (168 births); missing data for pre-pregnancy BMI, pregnancy weight gain, birthweight and lipid values (102 births); maternal age less than 19 or older than 44 years old (24 births); birthweight < 500g or > 7000g (0 birth). Finally, the study population was consisted of 1 410 singleton births of 705 women. No malignant tumor, chromosomal abnormalities, or metabolic diseases before pregnancy were recorded. And no one experienced serious infection, used tobacco, or consumed alcohol or drugs.

**Figure 1:**
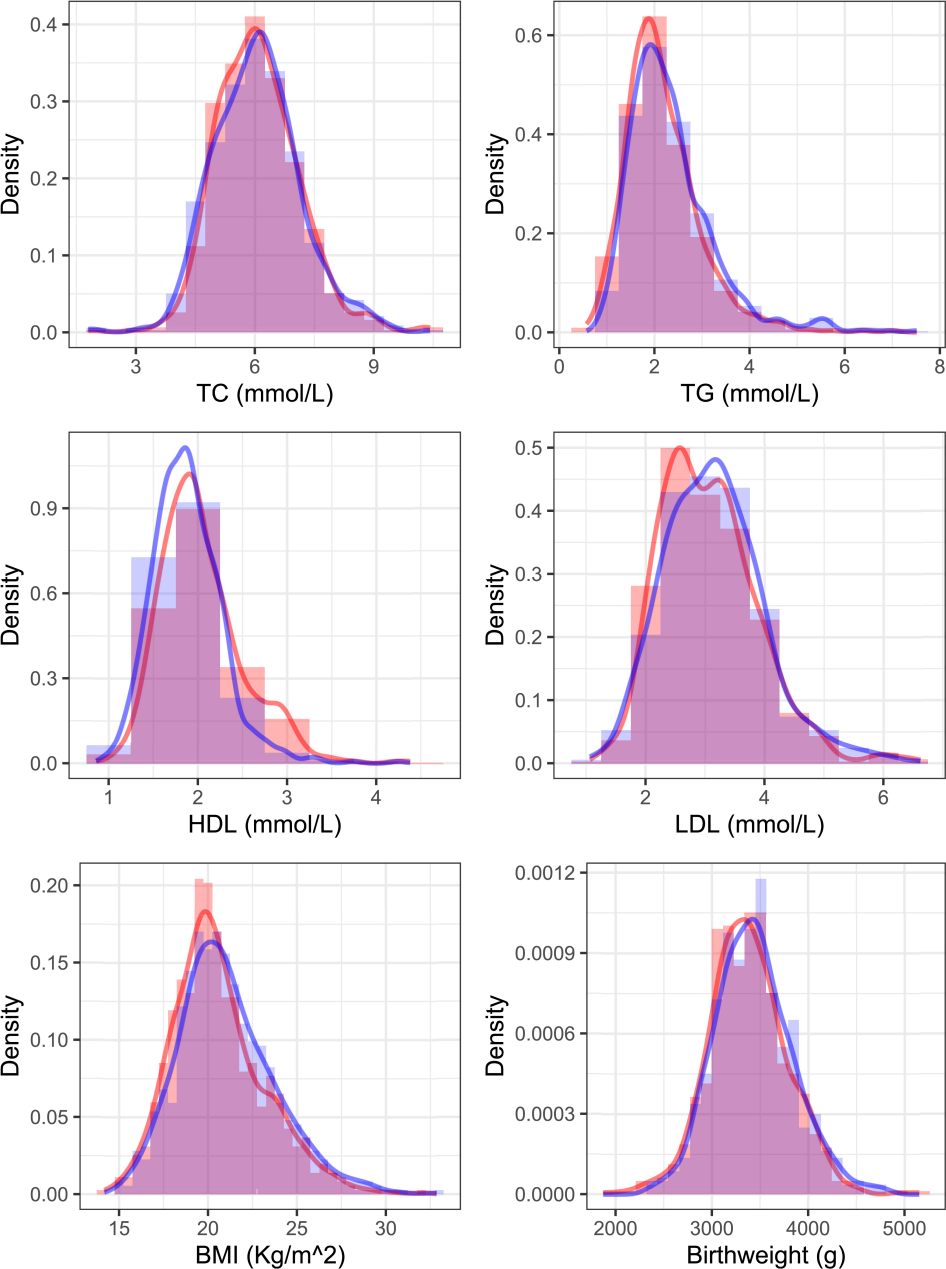
Distribution of 2^nd^-trimester serum lipids and birthweight of the first and second pregnancy. The red color shows the distribution curve in an older sibling and blue shows the next youngest sibling.

**Table 1** showed descriptive characteristics of mothers and infants in each pregnancy. All newborns included in our analysis had a mean (SD) birth weight of 3405 (407.50) g, and the gestational age at birth was 39.19 (1.12) weeks. In addition, 691 (49%) infants were boys. For maternal lipid values, we can see that TG in the second trimester and HDL-C values in the second and third trimesters have significant differences. **Figure 1** shows the distribution of maternal lipid values in the second trimester and birthweight of the first and second pregnancy.

**Table 1:** Overall characteristics of mothers and neonates in the first and second pregnancy.

Characteristics	First pregnancy	Second pregnancy	P value^†^
Maternal age (mean (SD))	28.41 (3.24)	30.39 (3.27)	<0.001
Maternal Education (%)			0.881
Under college	85 (12.10)	85 (12.10)	
College or equivalent	538 (76.30)	538 (76.30)	
Above college	82 (11.60)	82 (11.60)	
Gravidity (mean (SD))	1.56 (0.84)	2.65 (0.92)	<0.001
Parity (mean (SD))	1.04 (0.21)	2.04 (0.21)	<0.001
Maternal weight gain (mean (SD))	14.92 (4.16)	13.86 (3.74)	<0.001
Husband age (mean (SD))	29.92 (4.08)	32.01 (4.14)	<0.001
Gestational week (mean (SD))	39.43 (1.10)	38.95 (1.09)	<0.001
Maternal pre-pregnancy BMI (%)			0.014
<18.5	146 (20.70)	108 (15.30)	
≥25	45 (6.40)	61 (8.70)	
18.5-24.9	514 (72.90)	536 (76.00)	
Neonatal weight (mean (SD))	3376 (409)	3434 (404)	0.007
Neonatal gender (%)			0.002
Male	316 (44.80)	375 (53.20)	
Female	389 (55.20)	330 (46.80)	
2^nd^-trimesterTC (mean (SD))	6.07 (1.05)	6.05 (1.09)	0.755
2^nd^-trimester TG(mean (SD))	2.17 (0.79)	2.34 (0.91)	<0.001
2^nd^-trimester HDL-C(mean (SD))	2.04 (0.47)	1.88 (0.39)	<0.001
2^nd^-trimester LDL-C (mean (SD))	3.06 (0.82)	3.15 (0.84)	0.077
3^rd^-trimeste TC(mean (SD))	6.48 (1.27)	6.53 (1.23)	0.488
3^rd^-trimeste TG (mean (SD))	3.49 (1.73)	3.45 (1.73)	0.679
3^rd^-trimeste HDL-C(mean (SD))	1.89 (0.61)	1.81 (0.48)	0.007
3^rd^-trimeste LDL-C(mean (SD))	3.33 (0.96)	3.42 (1.01)	0.193

Data are mean (SD) or n (%).

^†^P values were calculated using one-way ANOVA (for continuous variables) or χ2 test (for categorical variables), and P<0.05 indicates that the mean values (for continuous variables) or proportions (for categorical variables) of a variable were significantly different between first and second pregnancy.

**Table 2**, using a mixed-effects model, showed the adjusted associations between maternal factors, lipid values and difference in birthweight. Gestational age, parity, neonatal gender, maternal pre-pregnancy BMI, maternal weight gain, and third-trimester TC were all positively related to higher birthweight. Third-trimester HDL-C was negatively associated with birthweight. Every unit increase in TC in the third trimester increases birthweight by 53.13 (14.32 ~91.94) g.

**Table 2:** Relationship between change in birthweight and maternal-fetal factors.

Maternal-fetal factors	Change in birthweight	se	t value	P value
(Intercept)	-2639.79	501.65	-5.26	<0.001
Maternal age	7.08	5.59	1.27	0.206
Maternal weight gain	27.36	3.06	8.95	<0.001
Maternal pre-pregnancy BMI	30.59	5.13	5.97	<0.001
Husband age	-7.13	4.31	-1.66	0.098
Parity	77.06	17.56	4.39	<0.001
Gestational week	120.59	11.64	10.36	<0.001
Neonatal gender-Male	157.55	22.49	7.00	<0.001
2^nd^-trimester TC	23.39	22.72	1.03	0.304
2^nd^-trimester TG	13.10	18.31	0.72	0.474
2^nd^-trimester HDL-C	-17.29	33.84	-0.51	0.609
2^nd^-trimester LDL-C	-31.18	29.95	-1.04	0.298
3^rd^-trimester TC	53.13	19.80	2.68	0.007
3^rd^-trimester TG	-0.34	9.77	-0.04	0.972
3^rd^-trimester HDL-C	-57.18	26.72	-2.14	0.033
3^rd^-trimester LDL-C	-40.57	25.69	-1.58	0.115

Change in birthweight per unit change in indicated variable. For example, infant birth weight increased by 27.36g per additional kg of weight gain during pregnancy after adjustment for the other variables.

**Figure 2:**
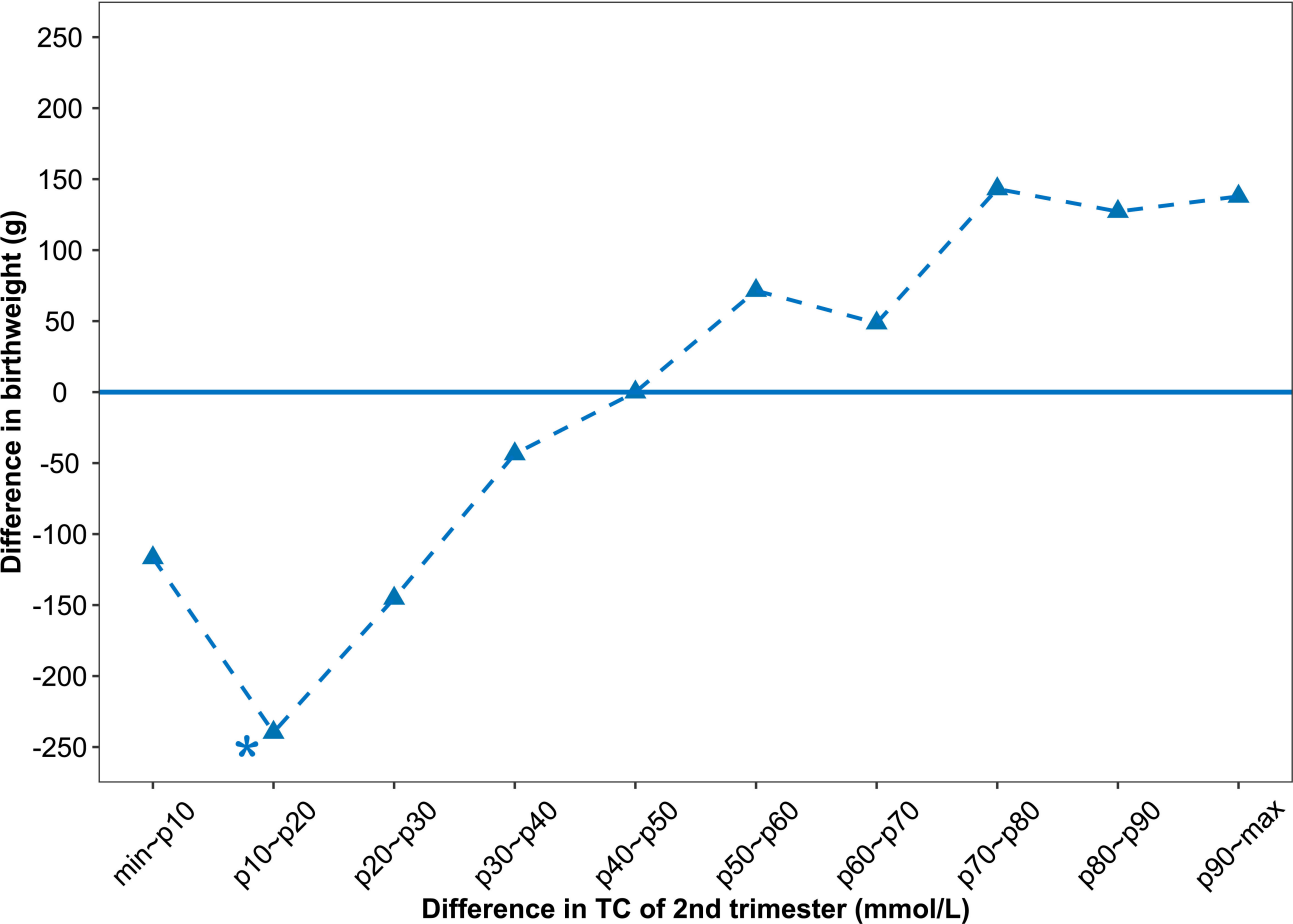
Associations between difference in 2^nd^-trimester TC and difference in birthweight. The difference in birthweight is relative to the reference group (infants of mothers whose 2^nd^-trimester TC increased by 40^th^-50^th^ percentile (-0.20 ~ -0.03 mmol/L). The variable values refer to the values of the second observed pregnancy minus the value of the first observed pregnancy for each mother.

Linear regression models involving maternal lipid values as a categorical variable were analyzed and only one significant result was found (shown in **Table 3**). The 10th -20th percentiles of the difference in 2^nd^-trimester TC were statistically significant compared with the 40th to 50th percentile. Infants of women whose 2^nd^-trimester TC increased by 10th-20th percentile (-0.92~-0.56 mmol/L) from 1st to 2nd pregnancy were 239.69 (62.32~ 417.06) g lighter at birth than were infants of women those of 40th-50th percentile (-0.20~-0.03 mmol/L). **Figure 2** plots coefficient estimates from the linear regression model (**Table 3**).

**Table 3:** Association between the difference in 2^nd^-trimester TC and difference in birthweight.

Difference in TC category	level	Difference in birthweight	LL	UL	P value
p40~p50 (Reference)	0	–	–	–	–
min~p10	-4	-116.74	-301.68	68.20	0.217
p10~p20	-3	-239.69	-417.06	-62.32	0.008
p20~p30	-2	-145.27	-325.30	34.76	0.115
p30~p40	-1	-43.56	-219.11	132.00	0.627
p50~p60	1	71.52	-101.17	244.22	0.417
p60~p70	2	48.63	-128.61	225.87	0.591
p70~p80	3	143.14	-37.62	323.89	0.121
p80~p90	4	127.19	-57.63	312.01	0.178
p90~max	5	137.86	-52.59	328.31	0.157

Difference in birthweight is the estimate of effect value of difference in TC between two deliveries on difference in birthweight. LL and UL are 95% CI of the estimate of effect value. min refers to the minimum value of difference in 2nd-trimester TC from 1st to 2nd pregnancy, which is -4.73 mmol/L, while max refers to the maximum value, which is 4.22 mmol/L. p10 to p90 refer to the percentile of difference in TC, which are -0.92, -0.56, -0.38, -0.20, -0.03, 0.15, 0.31, 0.53, 0.87mmol/L.

The model is adjusted for maternal age, parity, pre-pregnancy BMI, gestational weight gain, gestational age at birth, infant gender, the interval of two pregnancies, the difference in gestational age and differences in 2^nd^-trimesterTG, HDL-C and LDL-C.

## Discussion

Maternal lipid profiles have been associated with birthweight and adverse pregnancy outcomes. Our study, using singleton births from population-based data, provides evidence for a causal association that is independent of shared genes. We confirmed that the difference in TC in the second trimester was positively associated with the difference in birthweight in the two pregnancies of the same mother. We also noted that every unit increase in TC in the third trimester increases birthweight by about 50g. According to the literature available so far, this is the first study to show an independent association between maternal TC levels and birthweight using a within-family design to minimize confounding. In addition, we found that the 2^nd^ trimester TG value significantly increased and HDL-C value significantly decreased in the second pregnancy, and the corresponding neonatal birthweight significantly increased. Because birthweight predicts adult BMI (19–23), our study suggests that maternal dyslipidemia could program the fetus for an increased long-term risk of obesity-related diseases.

According to existing research, the relationship of maternal lipids and birthweight has been studied among different countries and races. In a recently published large-scale birth cohort study of Japanese mothers with singleton birth at term, maternal TC levels in midpregnancy, independent of other potential confoundings including prenatal weight gain and pre-pregnancy BMI, are significantly correlated with the birth of SGA or LGA (24). In rural India, undernourished pregnant women with normal glucose, neonatal birthweight was significantly associated with maternal blood glucose levels and lipid concentrations at 18 and 28 weeks. Similarly, linear correlation was found between maternal TC and birth size (25). One cohort study in the United State reported that low maternal TC in midpregnancy was associated with SGA (26). Our study is in line with the above studies and further confirms these findings independent of shared genes and environmental facts. The results may provide evidence for further research and potential mechanism.

In early pregnancy, maternal cholesterol is fundamental to hormonal and physical changes (27–34) and is associated with the development of both embryonic and placental tissues. Circulating cholesterol is transported to the embryo and could affect the placenta transport function by changing the placental cholesterol concentrations (35). As gestation progresses, maternal total cholesterol levels gradually increase and this physiologic hypercholesterolemia especially comes up in the second and third trimesters (36–39), which maintain pregnancy and fetal growth. Maternal TG, which cannot cross the placenta, is hydrolyzed to fatty acids by placental lipases and then taken up by placental trophoblasts, where they can be metabolized or transported to fetal circulation (40, 41). These fatty acids are thought to be substrates for fetal adipogenesis or provide the energy needed for fetal growth (42, 43). The fetus can derive lipid from both endogenous and exogenous pathways. The fetus can synthesize lipid through their own metabolism, which called the endogenous pathway. And lipid obtained from the yolk sac or placenta is called the exogenous pathway. Any factors affecting these two pathways of lipid access could cause abnormal fat accumulation within the fetus (27). Too high or low fat intake can both affect fetal growth and even adipose tissue development, adipocyte differentiation in offspring, which have been shown in previous animal studies (44, 45). This mechanism may confirm the “fetal origin hypothesis” and an increased risk of cardiovascular disease in the adult (46–48). In addition, decreased or increased maternal TC levels have been reported to be associated with abnormal birthweight and adverse pregnancy complications. In our study, the difference in TC levels between two pregnancies of the same pregnant woman was positively correlated with the difference in newborn birthweight, which is worthy of further exploring the related factors affecting birthweight in the metabolism pathway.

One interesting finding of our study was that maternal pre-pregnancy BMI and birthweight were significantly increased in the second pregnancy. A recent study showed that a higher maternal pre-pregnancy BMI was associated with altered maternal early-pregnancy nonesterified fatty acids (49). In the second pregnancy, the TG value was significantly increased and the HDL-C value was significantly decreased, which may declare a higher risk of dyslipidemia. Diet and lifestyle may cause these changes and influence the birthweight and metabolism of offsprings. As reported, metabolic disturbance of maternal TG and non-esterified fatty acid were correlated with excessive birthweight as Herrera et al. (4, 30). The Amsterdam Born Children and their Development cohort study focused on the association between early pregnancy maternal TG concentrations and adverse pregnancy outcomes including LGA, preterm birth, pregnancy-induced hypertension, and PE and discovered they were linearly correlated (50). This also provides a basis for predicting birthweight.

The highlight of our research is the providing of attractive evidence regarding the association of maternal TC concentrations and birthweight within the same mother. The interaction between glucose and lipid metabolism during pregnancy is still controversial. No within-family studies have been conducted investigating the association between maternal lipid and birthweight before, and less attention has been paid to TC levels. Our study design excluded more confounding factors and provides a good complement to existing research. Some guidelines have proposed the importance of prepregnancy lipid screening, but an unified standard is still lacking. Our study can provide a basis for the formulation of the reference range of blood lipid during pregnancy. In routine clinical practice, dietary interventions/daily activities can be managed by maternal lipid levels.

However, there are still some limitations. The primary limitation of the study was that it was a single-center cohort study and our sample size was not large enough. Further research should expand the sample size and conduct a multi-center and multi-region prospective investigation. There is an outlier and missing data in lipid values, we think this confounding would also be decreased by enlarging the sample size. Additionally, only second and third-trimester lipid values were included in our study. Maternal lipids concentrations before pregnancy and through the whole pregnancy should better to be collected. Thus, we can move the point of intervention forward and establish a prediction model. Our within-family design has effectively eliminated confounding by genetic and other relevant factors especially environmental factors. In addition, the information that could be cobfoundings about the difference in physical activities during the two pregnancies and family history of diabetes was not obtained Furthermore, there is no measure of neonatal body fatness so the authors are unable to say whether the difference in lipids between pregnancies influences somatic growth or adipose tissue deposition in the offspring.

In conclusion, the difference in third trimester TC was positively associated with the difference of birthweight in the two pregnancies of the same mother independent of other potential confounding factors, mainly for prenatal weight gain and pre-pregnancy BMI. Every unit increase in TC in the third trimester increases birthweight by 53.13 (14.32 ~91.94) g. Given the apparent associations, TC may be used to guide diet and predict birthweight combined with ultrasound and other indicators. Further studies can explore optimal maternal serum cholesterol levels during pregnancy to prevent adverse pregnancy outcomes and adult obesity related diseases.

## Data availability statement

The raw data supporting the conclusions of this article will be made available by the authors, without undue reservation.

## Ethics statement

Written informed consent was obtained from the individual(s) for the publication of any potentially identifiable images or data included in this article.

## Author contributions

QL and HH designed the study. QC was involved in the study design. MW and LQ enrolled the patients. HC analysed the data. QC and HC wrote the article. All other authors – FX, YJ, and ML critically reviewed and approved the final manuscript.

## Funding

This work was supported by Scientific Research Foundation of the National Health Commission (WKJ-ZJ-2126), National Key Research and Development Program (2021YFC2700700), Key Laboratory of Gamete and Reproductive Tract Abnormalities, National Health Commission(NHC-2020-2).

## Acknowledgments

The authors thank the Staff at the women’s hospital, Zhejiang University for technical assistance and facility support. We are also grateful to all the mothers and newborns that participated in the study and thank all the midwives and obstetricians who carefully gathered and recorded data.

## Conflict of interest

The authors declare that the research was conducted in the absence of any commercial or financial relationships that could be construed as a potential conflict of interest.

## Publisher’s note

All claims expressed in this article are solely those of the authors and do not necessarily represent those of their affiliated organizations, or those of the publisher, the editors and the reviewers. Any product that may be evaluated in this article, or claim that may be made by its manufacturer, is not guaranteed or endorsed by the publisher.
